# Microbial Metabolites: The Emerging Hotspot of Antiviral Compounds as Potential Candidates to Avert Viral Pandemic Alike COVID-19

**DOI:** 10.3389/fmolb.2021.732256

**Published:** 2021-09-07

**Authors:** Topu Raihan, Muhammad Fazle Rabbee, Puja Roy, Swapnila Choudhury, Kwang-Hyun Baek, Abul Kalam Azad

**Affiliations:** ^1^ Department of Genetic Engineering and Biotechnology, Shahjalal University of Science and Technology, Sylhet, Bangladesh; ^2^ Department of Biotechnology, Yeungnam University, Gyeongsan, South Korea; ^3^ Department of Genetic Engineering and Biotechnology, Jagannath University, Dhaka, Bangladesh

**Keywords:** antiviral, microbial metabolites, pandemic, SARS-CoV-2, COVID-19

## Abstract

The present global COVID-19 pandemic caused by the noble pleomorphic severe acute respiratory syndrome coronavirus 2 (SARS-CoV-2) has created a vulnerable situation in the global healthcare and economy. In this pandemic situation, researchers all around the world are trying their level best to find suitable therapeutics from various sources to combat against the SARS-CoV-2. To date, numerous bioactive compounds from different sources have been tested to control many viral diseases. However, microbial metabolites are advantageous for drug development over metabolites from other sources. We herein retrieved and reviewed literatures from PubMed, Scopus and Google relevant to antiviral microbial metabolites by searching with the keywords “antiviral microbial metabolites,” “microbial metabolite against virus,” “microorganism with antiviral activity,” “antiviral medicine from microbial metabolite,” “antiviral bacterial metabolites,” “antiviral fungal metabolites,” “antiviral metabolites from microscopic algae’ and so on. For the same purpose, the keywords “microbial metabolites against COVID-19 and SARS-CoV-2” and “plant metabolites against COVID-19 and SARS-CoV-2” were used. Only the full text literatures available in English and pertinent to the topic have been included and those which are not available as full text in English and pertinent to antiviral or anti-SARS-CoV-2 activity were excluded. In this review, we have accumulated microbial metabolites that can be used as antiviral agents against a broad range of viruses including SARS-CoV-2. Based on this concept, we have included 330 antiviral microbial metabolites so far available to date in the data bases and were previously isolated from fungi, bacteria and microalgae. The microbial source, chemical nature, targeted viruses, mechanism of actions and IC_50_/EC_50_ values of these metabolites are discussed although mechanisms of actions of many of them are not yet elucidated. Among these antiviral microbial metabolites, some compounds might be very potential against many other viruses including coronaviruses. However, these potential microbial metabolites need further research to be developed as effective antiviral drugs. This paper may provide the scientific community with the possible secret of microbial metabolites that could be an effective source of novel antiviral drugs to fight against many viruses including SARS-CoV-2 as well as the future viral pandemics.

## Introduction

**FIGURE 1:**
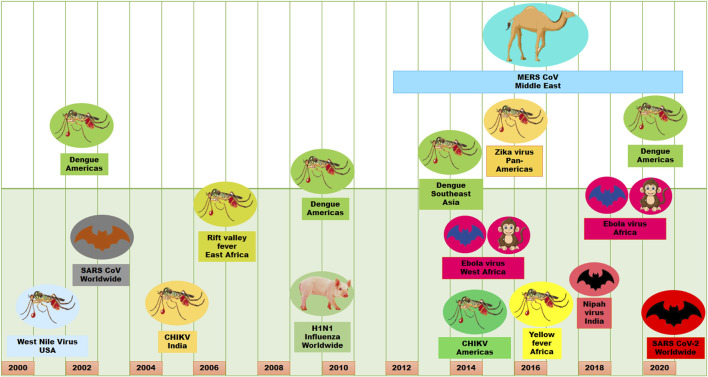
Viral outbreaks in the last 2 decades.

Viral infections are one of the major causes of morbidity and mortality in the world. It is very catastrophic due to the complexity, diversity, obligatory intracellular parasitic nature and pleomorphic character of viruses. These properties of viruses make it very difficult to counteract viral effects and transmission, which ultimately causes epidemics and/or pandemics (Graham et al., 2013; Meganck and Baric, 2021). Although the deadly influenza outbreak occurred in 1918, in the last 2 decades of the present century, there have been several viral epidemics or pandemics in humans (Figure 1). These viral epidemics or pandemics were caused with influenza A virus (H1N1), severe acute respiratory syndrome coronavirus (SARS-CoV), Middle East respiratory syndrome coronavirus (MERS-CoV), dengue virus (DENV), Zika virus (ZIKV), Ebola virus (EBOV), chikungunya virus (CHIKV), Henipavirus (HeV, NiV) and the recent severe acute respiratory syndrome coronavirus 2 (SARS-CoV-2) (Meganck and Baric, 2021). Moreover, human immunodeficiency virus (HIV) is life-threatening since its discovery in 1982. Some other viruses such as Crimean–Congo hemorrhagic fever virus, Herpes simplex virus, Hepatitis viruses, Rabies virus, Hantaviruses have caused outbreaks or have outbreak potential. Therefore, the increase of migration, global travel, and urbanization have made viruses outbreaks a crucial challenge for public health, especially when vaccines and antiviral therapies are still not available (Neiderud, 2015).

Viruses having a genome either RNA or DNA utilize the molecular apparatus of the host cells for their replication and cause several ailments (Tapparel et al., 2013; Cohen, 2016). Viral infections can be controlled by prophylactic strategy and/or drug therapy. However, for being obligatory intracellular parasite, most of the metabolic pathways involved in the viral replication are the same as in the host cells. From this point of view, it is difficult to design an appropriate treatment to attack the virus without triggering adverse events on the host. These aspects further highlight the main peculiarity of viruses (specificity, affinity, and self-defense mechanisms) and the difficulties of antiviral chemotherapy. Therefore, it is necessary to discover and identify new antiviral agents, which should possess primarily an adequate selectivity, power, *in vivo* stability profile and low toxicity (Akram et al., 2018).

Many natural and synthetic drugs having antiviral activity were considerably less effective when tested in virus-infected animal models (Martinez et al., 2015; Takizawa and Yamasaki, 2018; Mukherjee, 2019). Moreover, extraction of the natural products from the plants and the chemical synthesis of synthetic drugs have safety and economic concerns. Furthermore, conventional drugs become failed against viral infections and the onset of specific viral resistances against these drugs is a common phenomenon (Linnakoski et al., 2018; Mulwa and Stadler, 2018; Ma et al., 2020). Therefore, researchers need to search for alternative source of safe and economically cost-effective antiviral natural products. In this context, microbial metabolites might be a promising source of antiviral agents. Microorganisms are natural flora of the environment that play significant role in plenty of processes, and therefore, their metabolites have great potential to be used for antiviral treatment without severe side-effects (Cheung et al., 2014). In fact, microbial metabolites have already been a subject of intense research for the treatment of certain virus-mediated diseases (Berdy, 2005), and currently, there is an emerging trend in biotechnology for therapeutic applications of microbial metabolites as antiviral agents (Yasuhara-Bell et al., 2010a; Pham et al., 2019; Goris et al., 2021; Lobo-Galo et al., 2021). Several microbial metabolites have been demonstrated to offer promising antiviral activity against numerous DNA and RNA viruses (Tong et al., 2012; Linnakoski et al., 2018; Mulwa and Stadler, 2018). The whole world has been fighting against the current COVID-19 pandemic for more than one and a half years. As there is no newly developed specific approved drug, only repurposed drugs are used as the supportive treatment of the stormy COVID-19 caused by SARS-CoV-2 (Hakim et al., 2021), which has caused total death of 4,374,234 in the world as on August 15, 2021. Cases and death of COVID-19 is going on ceaselessly globally. As the trend of the history, more viral epidemics and/or pandemics may outbreak in the future. Therefore, it is essential to discover drugs with broad spectrum activity against SARS-CoV-2 including other catastrophic viruses. Screening and identification of natural compounds from microbial metabolites may be particularly important for drug discovery against the coronavirus alike SARS-CoV-2 as well as other viruses having potential outbreaks in the future.

This review focuses on microbial metabolites, which have shown activity against various viral pathogens. In addition, the current state of this research topic is briefly discussed, and gaps in the research are identified. Furthermore, the targets for antiviral therapeutic development and the advantages of microbial metabolites are briefly discussed. Finally, this review attempts to offer alternative conceptual framework for drug discovery for treatment of COVID-19 and alike future viral pandemics and/or epidemics.

## Targets of Microbial Metabolites for Therapeutic Development

**FIGURE 2:**
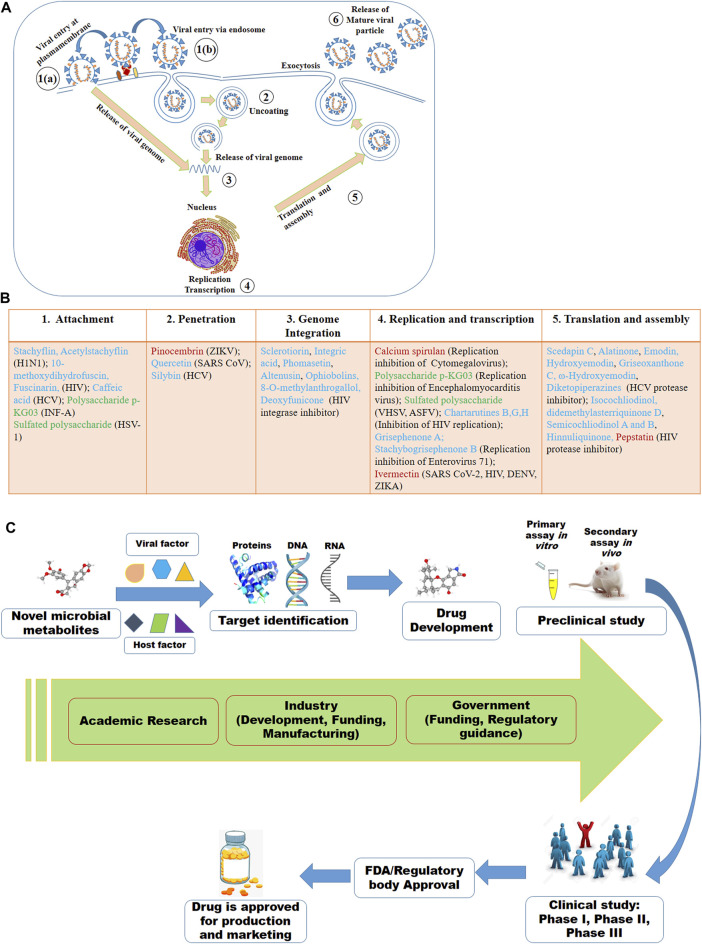
Viral lifecycle **(A)** and the proposed mode of actions of some of the antiviral microbial secondary metabolites (MSM) **(B)** listed in this review. The numbers in **(A)** denote the steps usually targeted by MSM. In **(B)**, some of the antiviral MSM inhibiting targeted stages of viral lifecycle are listed. Cyan, red and green colors indicate the metabolites isolated from fungi, bacteria and microalgae, respectively. **(C)** Antiviral drug development strategy based on the viral and host factors.

Despite of having different biology for infection, viruses share some basic steps for their replication (Figure 2A). The basic steps for viral replication include 1) viral attachment to host cells (host-viral interaction), 2) viral penetration into host cells, 3) viral uncoating into the cytoplasm, 4) viral genome replication and transcription, 5) viral protein translation and assembly, and 6) viral progeny release (Meganck and Baric, 2021). Due to having limited numbers of own coding genes, viruses must depend on the host machinery for accomplishment of viral lifecycle. The fundamental steps involved in viral lifecycle are associated with viral infection as well as pathogenesis and represent important targets for therapeutic development. The infection or the pathogenesis starts with the viral entry into the host cells (Ryu, 2017; Thaker et al., 2019). The prerequisite for viral entry is its binding on the cell surface. Viral proteins on the capsid or envelope interact with the specific receptor, which can be proteins, glycans and/or lipids in the host cell. For instance, the spike protein S of SARS-CoV-1 and SARS-CoV-2 interact with the angiotensin-converting enzyme 2 (ACE2) as the receptor expressed on the surface of the target cells (Lim et al., 2016; Fung and Liu, 2019; Chen et al., 2020; Hoffmann et al., 2020; Ou et al., 2020; Rahman et al., 2020; Walls et al., 2020; Yan et al., 2020). The interaction between the viral protein and host receptor facilitate the viral uptake often through endocytic pathways or through fusion at the plasma membrane (Millet and Whittaker, 2018; Milewska et al., 2020). Viruses escape the endosome by uncoating and the genomic material is released into the cytoplasm.

Replication of DNA viruses is performed by using DNA dependent DNA polymerase. DNA viruses can integrate their genomes into the host genome and cause recurrent problem. RNA viruses replicate their genomes either by RNA-dependent RNA synthesis, or by RNA-dependent DNA synthesis (reverse transcription) which is followed by DNA replication and transcription. The genetic material of single-stranded positive sense RNA (ssRNA+) viruses is like mRNA which is directly translated by the host cell. The negative sense RNA (ssRNA−) viruses carry RNA that is complementary to mRNA and must be turned into ssRNA+ using RNA polymerase before translation. All positive sense RNA viruses like poliovirus, hepatitis C virus, dengue virus, ZIKV, SARS-coronavirus can arrange specialized membranous structures by remodeling host membranes where the viral genome is replicated (Cameron et al., 2009; Paul and Bartenschlager, 2013). Due to lack of RNA polymerase proofreading ability, RNA viruses have very high rate of mutation compared to DNA viruses, which eventually renders enhanced virulence and evolvability (Duffy, 2018).

Although all viruses utilize the host apparatus system for translation, viral translation is regulated differently from the host cell (Jan et al., 2016). Viral proteins and genomic materials are assembled to form the virion. The final stage of viral replication is the release of the new virions produced in the host organism. The new virions are then able to infect nearby cells and repeat the replication cycle. Some viruses are released when the host cell dies, while other viruses without directly killing the cell can leave infected cells by budding through the membrane (Lodish et al., 2000; Risco et al., 2014). The essential molecular elements involved in each of these steps in the viral lifecycle can be targeted by microbial metabolites as therapeutics.

The microbial metabolites may target either the viral or the host factors that are associated with viral pathogenesis or the completion of the viral lifecycle or viral replication (Figure 2B). The viral factors might be viral proteins associated with the binding of viruses to cells, viral protease, viral translation or others (Anderson et al., 1996; Klemm et al., 2020; Chen C. C. et al., 2021). Host-factors might be receptor on the cell surface, endocytosis, host proteases and kinases, and others (Inoue et al., 2007; Ivanov, 2008; Raj et al., 2013; Zhou et al., 2015; Kalil et al., 2021). However, the viral and the host factors associated with the viral pathogenesis and its lifecycle or replication may vary based on the viruses even of the same family. For instance, while the spike protein S of SARS-CoV-1 and SARS-CoV-2 bind with the ACE2 receptor, the S protein of MERS-CoV binds to dipeptidyl peptidase 4 (DPP4) receptor (Raj et al., 2013; Lim et al., 2016; Hoffmann et al., 2020; Rahman et al., 2020; Walls et al., 2020; Yan et al., 2020). Here, viral S protein may serve as the drug target for all these three SARS viruses, however, ACE2 might be the target for the earlier two SARS viruses and the DPP4 might be for the MERS-CoV. Similarly, a serine protease named TMPRSS2 found to be essential for the activation of hemagglutinin (HA), the key step for initiating the viral infection by the H7N9 variant of H1N1, may be an important therapeutic target. The HA activation was failed in H7N9 virus when the TMRSS2 was knocked out in the mice (Tarnow et al., 2014).

Despite the viral life cycle, a number of factors regulate the host response towards certain viral infections (Fung and Liu, 2019; Azad et al., 2021; Hakim et al., 2021). The inaugural stages of diseases include the viral phase with the appearance of symptoms. However, with the progresses of the disease, the viral phase is replaced by the host inflammatory phase, which controls viral replication usually by damaging the host cells (Peiris et al., 2003). Antiviral therapeutics are active during the viral phase or viral life cycle after which these drugs become ineffective (Widagdo et al., 2017). Treatment options for controlling inflammatory damage during inflammatory phase usually include steroids as immunomodulatory and anti-inflammatory drugs (Yang J.-W. et al., 2020). In the ongoing pandemic, the hospitalized patients with COVID-19 are being treated with the corticosteroid dexamethasone (Group, 2021). Again baricitinib, a kinase inhibitor in the JAK/STAT signaling pathway, has been approved for COVID-19 treatment, which lowers cytokine release that is a hallmark in SARS-CoV-2 infection (Stebbing et al., 2020; Hakim et al., 2021; Kalil et al., 2021). Nevertheless, the interferon (IFN) alpha and beta activates the JAK/STAT signaling pathway that in turn triggers the synthesis of a number of antiviral gene products (Chiang and Liu, 2019). Therefore, any essential event involved in the viral phase and/or the host inflammatory phase might be an important target for treatment of the respective viral disease with microbial metabolites.

## Microbial Metabolites as Potential Antiviral Candidates

**FIGURE 3:**
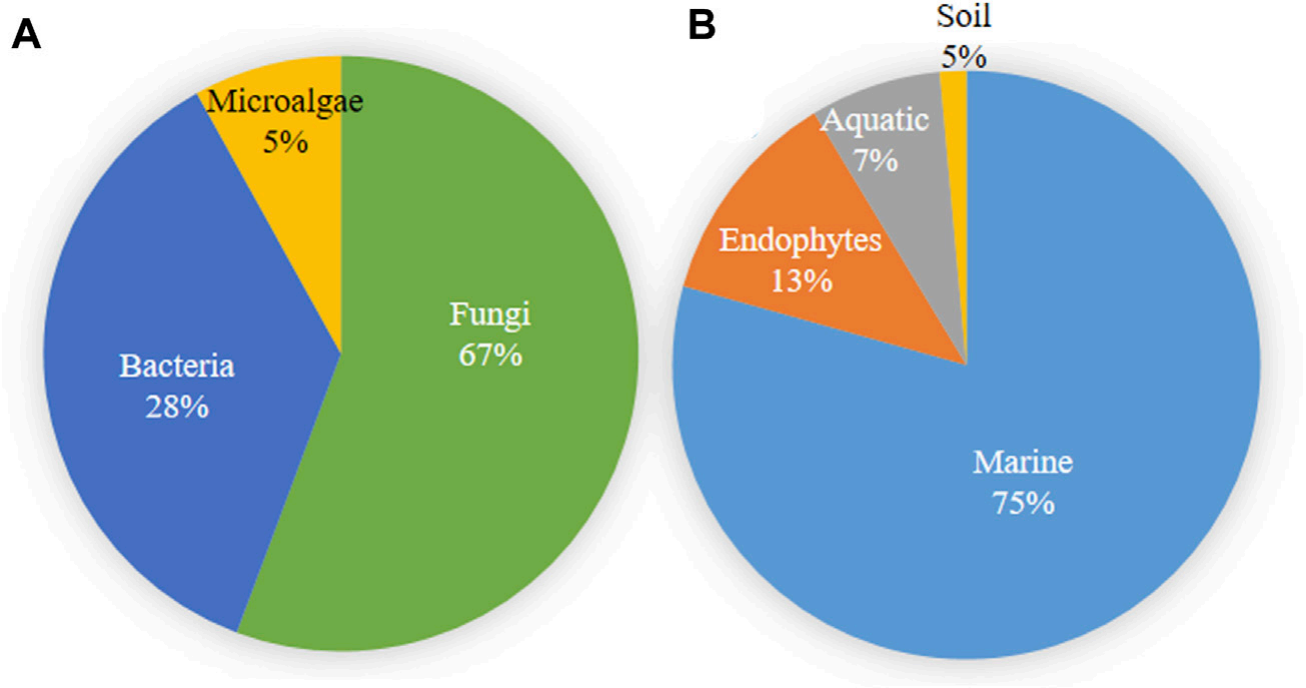
Microbial source of antiviral compounds **(A)** and the source of microorganisms producing antiviral compounds **(B)**.

**FIGURE 4:**
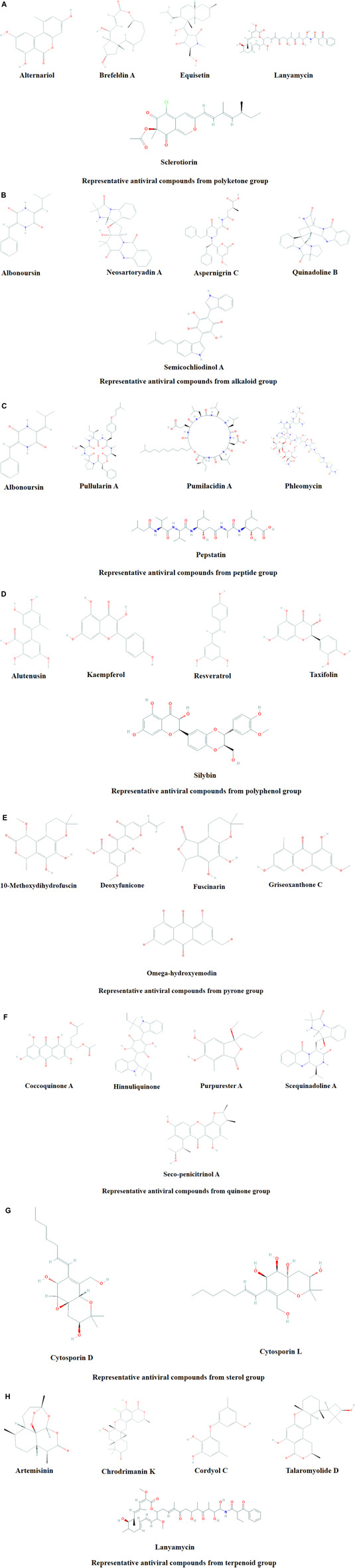
Representative antiviral compounds from polyketone **(A)**, alkaloid **(B)**, peptide **(C)**, polyphenol **(D)**, pyrone **(E)**, quinone **(F)**, sterol **(G)**, and terpenoid **(H)** groups.

Microbial metabolites are being used as important therapeutics for treatment of infections in health and agriculture arena (Demain, 2007; Raihan et al., 2021). For being advantageous over chemically synthesized and non-microbial natural products, research and development programs are continuously adopting approaches based on microbial products for the development of novel drugs. Microbial secondary metabolites (MSMs) have been being used as easy and reliable sources for the synthesis of new pharmaceuticals and therapeutics against different types of pathogens including viruses, bacteria, fungi and parasites (Demain, 2007; Selim et al., 2018). Many microorganisms such as bacteria, fungi, actinomycetes and microalgae from numerous sources have a variety of secondary metabolites like quinones, terpenoids, lignans, alkaloids, peptides, polysaccharides, lactones, polyketide, xanthone, ester, and so on having diverse antiviral activities (Selim et al., 2018; Pan et al., 2019). Several classes of such MSMs have been used as antiviral agents. From the literatures reported previously, only the antiviral metabolites from fungi, bacteria and microalgae have been listed in the present review (Tables 1–3). Fungi from different sources are the major reservoir of antiviral metabolites followed by bacteria and microalgae. Most of the MSMs were isolated from microorganisms of the marine source (Figure 3). The MSMs clusters to different groups (Figure 4) having different mechanism of actions against viruses. Although the mechanism of actions of most of the antiviral microbial metabolites are not yet elucidated, that of a few microbial metabolites has been reported (Tables 1–3). Elucidation of mode of actions and pharmacological properties of novel antiviral microbial bioactive metabolites may lead to the development of drugs for treating human diseases developed by catastrophic viral agents.

**TABLE 1:** Antiviral bioactive compounds isolated from fungi.

SL.	Fungi	Antiviral compounds	Group	Targeted Virus	Mechanism of inhibition	Source of the microbe	IC_50_/EC_50_ value	References
1.	*Penicillium sclerotiorum*	Sclerotiorin	Polyketone	HIV1	HIV-1 integrase and protease	Endophyte	14.5 and 62.7 µg/ml	Arunpanichlert et al. (2010)
2.	*Phomopsis* sp.	2-deoxy-4α-hydroxyoblongolide X	Polyketone	HSV1	NR	Endophyte	76 μM	Bunyapaiboonsri et al. (2010)
3.	*Xylaria mellisii*	Mellisol; 1,8- dihydroxynaphthol 1-O-α-glucopyranoside	Polyketone	HSV-1	NR	NR	10.50 and 8.40 μg/ml	Pittayakhajonwut et al. (2005)
4.	*Penicillium chrysogenum*	Sorbicatechol A and B	Polyketone	H1N1	NR	Marine	85 and 113 µM	Peng et al. (2014)
5.	*Penicillium* sp.	Brefeldin A	Polyketone	DENV, ZIKV, JEV	Dengue virus life cycle	NR	54.6 ± 0.9 nM	Raekiansyah et al. (2017)
6.	*Trichoderma* sp.	ZSU-H85 A	Polyketone	EV71	NR	NR	25.7 μM	Pang et al. (2018)
7.	*Fusaricum heterosporum*	Equisetin	Polyketone	HIV	NR	Marine	15 μM	Sims et al. (2005)
8.	*Pleospora tarda*	Alternariol; alternariol-9-methyl ether	Polyketone	HSV	Viral replication	Endophyte	13.5 and 21.3 µM	Selim et al. (2018)
9.	*Phoma* sp.	Phomasetin	Polyketone	HIV	HIV integrase	Marine	7–20 µM	Singh et al. (1999)
10.	*Aspergillus terreus*	12α-Dehydroxyisoterreulactone A; Arisugacin A; Isobutyrolactone II; Aspernolide A	Polyketone	HSV1	NR	Marine	16.4 ± 0.6, 6.34 ± 0.4, 21.8 ± 0.8 and 28.9 ± 0.8 µg/ml	Nong et al. (2014)
11.	*Ascomycetous* strain	Balticolid	Polyketone	HSV	Viral replication	Marine	0.45 µM	Shushni et al. (2011)
12.	*Ascomycetous strain*	Balticols A–F	Polyketone	H1N1, HSV	NR	Marine	1, 1, 1, 0.1, 0.01, 0.1 µg/ml (HSV)	Shushni et al. (2009)
13.	*Trichodesmium erythraeum*	Debromoaplysiatoxin; Anhydrodebromoaplysiatoxin; 3-Methoxydebromoaplysiatoxin	Polyketone	CHIKV	NR	Marine	^a^1.3, 22.3, 2.7 μM	Gupta et al. (2014)
14.	*Aspergillus terreus*	Pulvic acid; Isoaspulvinone E; Aspulvinone E	Polyketone	H1N1	NR	Soil	32.3; 56.9 and 29.1 µg/ml	Gao et al. (2013)
15.	*Pestalotiopsis* sp.	Pestalotiolide A	Polyketone	EV71	NR	Marine	27.7 µM	Jia et al. (2015)
16.	*Truncatella angustata*	Truncateol M	Polyketone	H1N1	NR	Marine	8.8 µM	Zhao et al. (2015)
17.	*Penicillium* sp.	Coniochaetone J	Polyketone	H1N1	NR	Marine	81.6 µM	Liu et al. (2017a)
18.	*Spiromastix* sp.	Spiromastilactones B, D–G, I–J and L	Polyketone	H1N1	NR	Marine	16.2 ± 0.6, 27.6 ± 0.4, 6.0 ± 0.2, 11.4 ± 1.3, 30.7 ± 1.7, 74.9 ± 4.9, 38.2 ± 2.1 and 22.6 ± 0.9 µM	Niu et al. (2016)
19.	*Streptomyces* sp.	Wailupemycin J; R-Wailupemycin K; Deoxyenterocin	Polyketone	H1N1	NR	Marine	NR	Liu et al. (2017b)
20.	*Streptomyces koyangensis*	Neoabyssomicin D	Polyketone	HSV	NR	Marine	NR	Huang et al. (2018)
21.	*P. chrysogenum*	Penicitrinone F	Polyketone	EV71	NR	Marine	14.50 μM	Chen et al. (2017)
22.	*Fusarium* sp.	Isochaetochromin D1	Polyketone	HIV	NR	NR	NR	Singh et al. (2003a)
23.	*Penicillium hesseltinei*	Hesseltin A	Polyketone	HSV-1	NR	NR	NR	Phipps et al. (2004)
24.	*Cladosporium sphaerospermum*	Cladosin C	Polyketone	H1N1	NR	Marine	276 µM	Wu et al. (2014)
25.	*Truncatella angustata*	Truncateol C,E,O,P	Polyketone	H1N1, HIV-1	NR	Marine	55, 63.5, 30.4 ± 0.4 µM (H1N1) and 39.0 ± 1.2, 16.1 ± 0.7 µM (HIV)	Zhao et al. (2015); Zhao et al. (2018a)
26.	*Phomopsis* sp.	2-deoxy4α hydroxyoblongolide X	Polyketone	HSV-1	NR	Endophyte	76 μM	Debbab et al. (2011)
27.	*Aspergillus sydowii* and *Penicillium citrinum*	Penicitrinol L	Polyketone	H5N1	NR	Marine	41.5 µM	Yang et al. (2018)
28.	*Aspergillus* sp.	6-O-demethylmonocerin; Monocerin	Polyketone	H1N1	NR	Marine	172.4 and 175.5 µM	Kong et al. (2015)
29.	*Aspergillus* sp.	Asteltoxins E, F	Polyketide	H3N2, H1N1	NR	Marine	6.2 ± 0.08 and 8.9 ± 0.3 μM (H3N2) 3.5 ± 1.3 μM (H1N1 by F)	Tian et al. (2016)
30.	*Pullularia* sp.	Pullularin A	Peptide	HSV1	NR	Endophyte	3.3 μg/ml	Isaka et al. (2007)
31.	*Nigrospora* sp.	Alternariol; 4-hydroxyalternariol-9-methyl ether	Peptide	HSV	NR	Endophyte	13.5 and 21.3 μM	He et al. (2012)
32.	*Scytidium* sp*.*	Halovir A-E	Peptide	HSV1, HSV2	NR	Marine	^a^1.1, 3.5, 2.2, 2; 3.1 µM	Rowley et al. (2004)
33.	*Streptomyces* sp.	(3Z,6Z)-3-(4-hydroxybenzylidene)-6-isobutylidenepiperazine-2,5-dione; (3Z,6S)-3-benzylidene-6-isobutylpiperazine-2,5-dione; Albonoursin	Peptide	H1N1	NR	Marine	41.5 ± 4.5, 28.9 ± 2.2 and 6.8 ± 1.5 µM	Wang et al. (2013)
34.	*Aspergillus terreus*	Asperterrestide A	Peptide	H1N1 and H3N2	NR	Marine	20.2 and 0.41 µM (H1N1 and H3N2)	He et al. (2013)
35.	*Fusarium* sp.	Sansalvamide A	Peptide	MCV	MCV topoisomerase	Marine	124 µM	Hwang et al. (1999)
36.	*Pestalotiopsis* sp.	Pestaloxazine A	Peptide	EV71	NR	Marine	14.2 ± 1.3 μM	Jia et al. (2015)
37.	*Aspergillus versicolor*	Diketopiperazines (DKPs)	Peptide	HCV	HCV protease	Marine	8.2 μg/ml	Ahmed et al. (2017)
38.	*Eutypella* sp.	Eutypellazines A–L	Peptide	HIV	NR	Marine	14.8 ± 1.2, 11.5 ± 0.8, 10.7 ± 1.3, 8.5 ± 0.5, 3.2 ± 0.4, 16.6 ± 0.5, 18.2 ± 1.3, 13.3 ± 0.6, 6.7 ± 2.1, 4.9 ± 1.1, 5.8 ± 0.7 and 5.9 ± 0.9 µM	Niu et al. (2017)
39.	*Eurotium rubrum*	Rubrumlines A–O	Peptide	H1N1	Hemagglutinin	Marine	NR	Chen et al. (2015)
40.	*Aspergillus flavipes*	Aspochalasin L	Peptide	HIV	Viral replication	Soil	71.7 µM	Rochfort et al. (2005)
41.	*Aspergillus niger*	Malformin C	Peptide	HIV	NR	Marine	1.4 ± 0.06 µM	Zhou et al. (2016)
42.	*Hypocladium inflatum gams*	Cyclosporine A	Peptide	HCV	Viral protein folding	NR	NR	Watashi et al. (2003)
43.	*Simplicillium obclavatum*	Simplicilliumtide J; Verlamelin A,B	Peptide	HSV	NR	Marine	14.0, 16.7, and 15.6 µM	Liang et al. (2017)
44.	*Acremonium persicinum*	Acremonpeptides A-B; Al (III)-acremonpeptide D	Peptide	HSV	NR	Marine	^a^16, 8.7, and 14 µM	Luo et al. (2019)
45.	*Aspergillus* sp.	Aspergillipeptides D-E	Peptide	HSV	NR	Marine	9.5 and 19.8 µM	Ma et al. (2017)
46.	*Aspergillus sydowii*	Diorcinol, CordyolC	Terpenoid	H3N2	NR	Marine	66.5, 78.5 µM	Wang et al. (2014)
47.	*Aspergillus ochraceus*	6β,9α-dihydroxy-14-p-nitrobenzoylcinnamolide	Terpenoid	H3N2, hEV71	NR	Marine algae	17.0 μM (H3N2)	Fang et al. (2014)
48.	*Penicillium camemberti*	Indole diterpenoids; Emindole SB; 21-Isopentenylpaxilline; Paspaline; Paxilline	Terpenoid	H1N1	NR	Marine	I28.3, 38.9, 32.2, 73.3, 34.1, 26.2, 6.6, 77.9, and 17.7 µM	Fan et al. (2013)
49.	*Xylaria* sp.	Integric acid	Terpenoid	HIV	HIV integrase	Marine	10 µM	Singh et al. (1999)
50.	*Saccharomyces cerevisiae*	Betulinic acid	Terpinoids	HIV	Viral release inhibition	GMM	NR	Huang et al. (2019a)
51.	*Yarrowia lipolytica*	Betulinic acid	Terpinoids	HIV	Viral release inhibition	GMM	NR	Sun et al. (2019)
52.	*S. cerevisiae*	Glycyrrhetinic acid	Terpinoids	HBV, HIV	NR	GMM	NR	Wang et al. (2019)
53.	*S. cerevisiae*	Oleanolic acid	Terpinoids	HCV	Inhibition in genome replication and transcription	GMM	NR	Zhao et al. (2018b)
54.	*S. cerevisiae*	Artemisinin	Terpinoids	HBV, HCV	NR	GMM	NR	Paddon et al. (2013)
55.	*Aspergillus sydowii*	(Z)-5-(Hydroxymenthyl)-2-(6′)-methylhept-2′-en-2′-yl)-phenol	Terpenoid	H3N2	NR	Marine	57.4 µM	Wang et al. (2014)
56.	*Aspergillus ochraceopetaliformis*	Ochraceopone-A; Isoasteltoxin and asteltoxin	Terpenoid	H1N1, H3N2	HCV protease	Marine	20.0/12.2 ± 4.10, 0.23 ± 0.05/0.66 ± 0.09, and 0.54 ± 0.06/0.84 ± 0.02 μM (H1N1/H3N2)	Wang et al. (2016)
57.	*Talaromyces* sp.	Talaromyolide D	Terpenoid	PRV	NR	Marine	3.35 µM	Cao et al. (2019)
58.	*Stachybotrys chartarum*	Stachybonoid A	Terpenoid	DENV	NR	Marine	NR	Zhang et al. (2017)
59.	*Neosartorya* sp.	Ophiobolins	Terpenoid	HIV1	HIV-1 integrase	NR	6.7 µM	Singh et al. (2003b)
60.	*Penicillium* sp.	Chrodrimanin K; Chrodrimanin N; 3-Hydroxypentacecilide A	Terpenoid	H1N1	NR	Marine	74, 58, and 34 µM	Kong et al. (2017)
61.	*Stachybotrys* sp.	Stachybogrisephenone B	Pyrone	EV71	Replication of EV-71	Marine	30.1 µM	Qin et al. (2015)
62.	*Stachybotrys* sp.	Stachyflin; Acetylstachyflin	Alkaloid	H1N1	Fusion of viral envelope and endosome	Marine	0.003 µM	Minagawa et al. (2002)
63.	*Cladosporium* sp.	Norquinadoline A; Oxoglyantrypine; Deoxynortryptoquivaline; Quinadoline B; Deoxytryptoquivaline; Tryptoquivaline	Alkaloid	Influenza virus A	NR	Soil	82, 85, 82, 87, 85, 89 µM	Peng et al. (2013)
64.	*Stachybotrys chartarum*	Chartarutines B,G,H	Alkaloid	HIV1	Viral replication	Marine	4.9, 5.57, 5.58 µM	Li et al. (2014)
65.	*Penicillium raistrickii*	Raistrickindole A; raistrickin	Alkaloid		HCV	Marine	^a^5.7 and 7.0 µM	Li et al. (2019a)
66.	*Neosartorya udagawae*	Neosartoryadins A-B	Alkaloid	H1N1	NR	Endophyte	66 and 58 µM	Yu et al. (2016)
67.	*Chrysosporium merdarium*	Semicochliodinol A and B	Alkaloid	HIV	HIV protease		0.17 μM	Loya et al. (1998)
68.	*Penicillium* sp.	Trypilepyrazinol, 3β-hydroxyergosta-8,14,24 (28)-trien-7-one	Alkaloid	HIV, HCV, H1N1	NR	Marine	4.6 (HIV) and 7.7 µM (HCV), 3.5 µM for another (HIV)	Li et al. (2019b)
69.	*Aspergillus niger*	Aspernigrin C	Alkaloid	HIV	NR	Marine	4.7 ± 0.4 µM	Zhou et al. (2016)
70.	*Trichobotrys effuse*	Trichobotrysins A, B, D	Alkaloid	H3N2, H1N1	NR	Marine	3.08, 9.37 and 3.12 µM	Sun et al. (2015b)
71.	*Scedosporium apiospermum*	Scedapin C	Alkaloid	HCV	HCV protease	Marine	^a^110.35 µM	Huang et al. (2017a)
72.	*Penicillium* sp.	(‒)-2′R-1-hydroxyisorhodoptilometrin; methyl 6,8-dihydroxy-3-methyl-9-oxo-9H-xanthene-1-carboxylate	Quinone	HBV	NR	Marine	NR	Jin et al. (2018)
73.	*Nocardia alba* KC710971	(Z)-1-((1-hydroxypenta-2,4-dien-1-yl)oxy)anthracene-9,10-dione	Quinone	NDV	NR	Marine	NR	Janardhan et al. (2018)
74.	*P. purpurogenum*	Purpurquinone B; Purpurquinone C; Purpurester A	Quinone	INF	NR	Marine	61.3, 64, 85.3 µM	Wang et al. (2011)
75.	*Nigrospora* sp.	6-O-demethyl-4-dehydroxyaltersolanol A	Quinone	H1N1	NR	Endophyte	NR	Zhang et al. (2016)
76.	*Penicillium chrysogenum*	Alatinone; Emodin; Hydroxyemodin	Quinone	HCV	HCV protease	Marine	NR	Hawas et al. (2013)
77.	*Chaetomium* sp.	Isocochliodinol; didemethylasterriquinone D	Quinone	HIV	HIV protease	NR	NR	Sekita, (1983)
78.	*Alternaria tenuissima*	Altertoxins I-III; V-VI	Quinone	HIV	Viral replication	Endophyte	NR	Bashyal et al. (2014); Stack et al. (1986)
79.	*Dichotomomyces cejpii*	Scequinadoline A	Quinone	DENV	NR	Marine	^a^128.60 µM	Wu et al. (2018)
80.	*Aspergillus versicolor*	Aspergilols H-I; Coccoquinone A	Quinone	HSV	NR	Marine	^a^4.68, 6.25 µM	Huang et al. (2017b)
81.	NR	Hinnuliquinone	Quinone	HIV1	HIV-1 protease	Endophyte	NR	Singh et al. (2004)
82.	*Stachybotrys* sp.	Grisephenone A; Stachybogrisephenone B; 3,6,8-Trihydroxy-1-methylxanthone	Pyrone	EV71	Replication of EV-71	Marine	50, 30.1, 40.3 µM	Qin et al. (2015)
83.	*Aspergillus iizukae*	Methyl-(2-chloro-l,6-dihydroxy-3-methylxanthone)-8-carboxylate; methyl-(4-chloro-l,6-dihydroxy-3-methylxanthone)-8-carboxylate; methyl-(4-chloro-6-hydroxy-1-methoxy-3-methylxanthone)-8-carboxylate; methyl-(6-hydroxy-1-methoxy-3-methylxanthone)-8-carboxylate; 4-chloro-1,6-dihydroxy-3-methylxanthone-8-carboxylic acid; 2,4-dichloro-1,6-dihydroxy-3-methylxanthone-8-carboxylic acid	Pyrone	HSV-1, HSV-2, H1N1	NR	Marine	NR	Kang et al. (2018)
84.	*Fusarium equiseti*	Griseoxanthone C; ω-Hydroxyemodin	Pyrone	HCV	HCV protease	Marine	NR	Hawas et al. (2016)
85.	*Oidiodendron griseum*	10-methoxydihydrofuscin; fuscinarin	Pyrone	HIV	Block the HIV entry	Soil	NR	Yoganathan et al. (2003)
86.	*Penicillium* sp.	Deoxyfunicone	Pyrone	HIV1	HIV-1 integrase	NR	11–19 µM	Singh et al. (2003a)
87.	*Cladosporium* sp.	3α-hydroxy-7-ene-6,20-dione	Sterol	RSV	NR	Marine	0.12 µM	Yu et al. (2018)
88.	*Cladosporium* sp.	Cladosporisteroid B	Sterol	H3N2	NR	Marine	16.2 µM	Pang et al. (2018)
89.	*Penicillium* sp.	3β-hydroxyergosta-8,14,24 (28)-trien-7-one	Sterol	HIV, H1N1	NR	Marine	3.5 µM (HIV); 0.5 µM (H1N1)	Li et al. (2019a)
90.	*Eutypella* sp.	Cytosporin L, D	Sterol	RSV	NR	Marine	72.01 µM	Liao et al. (2017)
91.	*Fusarium oxysporum*	Podophyllotoxin	Lignan	HIV	HIV infection	Endophyte	NR	Kour et al. (2008)
92.	*Exophiala pisciphila*	2,4-dihydroxy alkyl benzoic acid	Polyphenol	HIV	Strand transfer reaction	NR	68 µM	Ondeyka et al. (2003)
93.	*Talaromyces flavus*	Altenusin	Polyphenol	HIV1	HIV-1 integrase	NR	11–19 µM	Singh et al. (2003a)
94.	*Fusarium incarnatum*	NA255	Polyphenol	HCV, HBV, HNV	Disrupts HCV replication complex	NR	NR	Sakamoto et al. (2005)
95.	*Aspergillus candidus*	Terphenyllin and 3-hydroxyterphenyllin	Polyphenol	HIV1	HIV-1 integrase	NR	11–19 µM	Singh et al. (2003b)
96.	*Y. lipolytica*	Naringenin	Polyphenol	HCV	NR	GMM	NR	Palmer et al. (2020)
97.	*S. cerevisiae*	Silybin	Polyphenol	HCV	Inhibit of penetration	GMM	NR	Yang, et al., (2020c)
98.	*Y. lipolytica*	Taxifolin	Polyphenol		NR	GMM	NR	Lv et al. (2020)
99.	*S. cerevisiae*	Kaempferol	Polyphenol	EV71	Inhibition in translation and assembly step	GMM	NR	Lyu et al. (2019)
100.	*S. cerevisiae*	Quercetin	Polyphenol	SARS-CoV	Inhibition in penitration	GMM	NR	Rodriguez et al. (2017)
101.	*S. cerevisiae*	Caffeic acid	Polyphenol	HCV	Inhibition in attachment	GMM	NR	Liu et al. (2019a)
102.	*S. cerevisiae*	Resveratrol	Polyphenol	RSV	NR	GMM	NR	Li et al. (2016)
103.	*Y. lipolytica*	Violacein	NR	HSV	NR	GMM	NR	Gu et al. (2020)
104.	*S. cerevisiae*	p-Coumaric acid	NR	ADV, HSV	NR	GMM	NR	Liu et al. (2019b)
105.	*Pestalotiopsis vaccinii*	Vaccinol J	NR	EV71	NR	Marine	30.7 µM	Wang et al. (2017)
106.	*Myriococcum albomyces*	Myriocin	NR	HCV, HBV, HNV	Propagation of HCV and HBV	NR	NR	Kluepfel et al. (1972)
107.	*Cylindrocarpon ianthothele*	8-O-methylanthrogallol	NR	HIV1	HIV-1 integrase	Lab	6 μM	Singh et al. (2003a)
108.	*Penicillium multicolor*	Isochromophilones I-II	NR	HIV	HIV-1 entry	NR	6.6 and 3.9 μM	Matsuzaki et al. (1995)
109.	*Penicillium islandicum*	(+)-rugulosin	NR	HIV1	HIV-1 integrase	NR	11–19 μM	Singh et al. (2003b)
110.	*Streptomyces koyangensi*	(4S)-10-hydroxy-10-methyl-11-oxo-dodec-2-en-1,4-olide	NR	HSV	NR	Marine	^a^25.4 µM	Huang et al. (2019b)
111.	*Chaetomium globosum*	Tetramic acid	NR	HIV	Chemokine receptor-5	NR	8.6 μM	Yang et al. (2006)
112.	*Fusarium oxysporum*	H1-A	NR	HCV	HCV protease	NR	NR	Yang et al. (2016)
113.	*Periconia* sp.	Pericoannosin A	NR	HIV	NR	Endophyte	69.6 µM	Zhang et al. (2015)
114.	*Aspergillus terreus*	Rubrolide S	NR	H1N1	NR	Marine	87.1 µM	Zhu et al. (2014)
115.	*Emericella* sp.	Emermidine A, B	NR	H1N1	NR	Endophyte	42.07 and 62.05 µg/ml	Zhang et al. (2011)
116.	*Pestalotiopsis fici*	Chloropupukeanolides	NR	HIV	HIV-1 replication	Endophyte	6.9 μM	Liu et al. (2011)
117.	*Cytonaema* sp.	Cytonic acid A, B	NR	hCMV	hCMV protease	Endophyte	43, 11 µmol	Guo et al. (2000)
118.	*Pestalotiopsis theae*	Pestalotheol C	NR	HIV1	NR	Endophyte	^a^16.1 µM	Li et al. (2008)

hCMV, human cytomegalovirus; HIV, human immunodeficiency virus; H1N1, Influenza A virus subtype H1N1; HSV, herpes simplex virus; DENV, dengue virus; hEV71, enterovirus 71; H3N2, Influenza A virus subtype H3N2; ZIKV, Zika virus; JEV, Japanese encephalitis virus; RSV, respiratory syncytial virus; HBV, hepatitis B; HCV, hepatitis C; WEEV, western equine encephalitis virus; PRV, pseudorabies virus; NR, not reported; GMM, genetically modified microorganism.

a Indicates EC_50_ value of the compound.

**TABLE 2:** Antiviral bioactive compounds isolated from bacteria and cyanobacteria.

SL.	Microorganism	Antiviral compounds	Group	Targeted virus	Mechanism of inhibition	Source of the microbe	IC_50_/EC_50_ value	References
1.	*Amycolatopsis orientalis*	Quartromicin	Peptide	HIV, HSV1, H1N1	NR	Soil	11–92 μ/ml (HSV1), 6.8–100 μ/ml (H1N1)	Tsunakawa et al. (1992)
2.	*Myxococcus stipitatus*	Phenalamide	Peptide	HIV-1	NR	NR	NR	Jurkiewicz et al. (1992)
3.	*Aetherobacter*	Aetheramides A and B	Peptide	HIV	NR	NR	0.015 μM	Plaza et al. (2012)
4.	*Bacillus pumilus*	Pumilacidins A-G	Peptide	HSV1	NR	Soil	NR	Naruse et al. (1989)
5.	*Actinomycetes*	Antipain, Elastatinal	Peptide	Polio virus	Poliovirus protease	NR	300 μM (Antipain) 250 μM (Elastatinal)	Molla et al. (1993); Belov et al. (2004)
6.	*Streptomyces* sp.	Phleomycin	Peptide	Polio virus	NR	NR	NR	Koch, (1971)
7.	*Streptomyces roseus*	Leupeptin	Peptide	Marburg virus	Host proteases	NR	NR	Gnirss et al. (2012)
8.	*Streptomyces* sp.	Pepstatin	Peptide	HIV	HIV protease	NR	25 nM	Richards et al. (1989); Roberts et al. (1990)
9.	*Nostoc ellipsosporum*	Cyanovirin-N	Peptide	HIV	Inhibition through binding to envelope protein gp120	Marine	0.3–395.5 nM	Botos et al. (2002)
10.	*Scytonema varium*	Scytovirin	Peptide	HIV	Inhibition through binding to viral coat proteins gp120, gp160, and gp41	NR	0.3–22 nM	Bokesch et al. (2003)
11.	*Spirulina platensis*	Allophycocyanin	Peptide	Enterovirus 71	Delay viral RNA synthesis	Marine	0.045 ± 0.012 μM	Shih et al. (2003)
12.	NR	Macrolactin A	Polyketone	NR	HIV replication	Marine	NR	Gustafson et al. (1989)
13.	*Sorangium cellulosum*	Sulfangolid C; soraphen F; spirangien B; epothilon D	Polyketones	HIV	Acetyl-CoA carboxylate transferase	NR	^a^16–50 nM.	Martinez et al. (2013)
14.	*Myxococcus stipitatus*	Rhizopodin	Polyketone	HIV	NR	NR	NR	Martinez et al. (2013)
15.	*Streptomyces koyangensis*	(4S)-10-hydroxy-10-methyl-11-oxo-dodec-2-en-1,4-olide	Polyketone	HSV	NR	Marine	^a^25.4 mM	Huang et al. (2019a)
16.	*Sorangium cellulosum*	Lanyamycin	Polyketone	HCV	NR	NR	NR	Gentzsch et al. (2011)
17.	*Streptomyces* sp.	Wailupemycin J; R-wailupemycin K; Deoxyenterocin	Pyrone, Polyketone	INF	NR	NR	NR	Liu et al. (2017b)
18.	*Streptomyces koyangensis*	Neoabyssomicin D	Polyketone	HSV	NR	Marine	NR	Huang et al. (2018)
19.	*Streptomyces youssoufiensis*	Violapyrones (VLPs) Q–T	Pyrone	H1N1, H3N2	NR	NR	58.8, 64.9, 30.6, 72.8 μM (H1N1) and 95, 63.9, 45.3, 72.8 μM (H3N2)	Hou et al. (2018)
20.	*Streptomyces puniceus*	Clazamycin	Alkaloid	HSV	NR	NR	NR	Dolak and DeBoer, (1980)
21.	*Streptomyces* sp.	(3Z,6Z)-3-(4-hydroxybenzylidene)-6-isobutylidenepiperazine-2,5-dione; (3Z,6S)-3-benzylidene-6-isobutylpiperazine-2,5-dione; Albonoursin	Alkaloid	H1N1	NR	Marine	41.5 ± 4.5 µM, 28.9 ± 2.2 µM, 6.8 ± 1.5 µM, respectively	Wang et al. (2013)
22.	*Streptomyces fradiae*	9(10H)-Acridanone	Alkaloid	WSSV	NR	Marine	NR	Manimaran et al. (2018)
23.	*Dichothrix baueriana*	β-carbolines and bauerines A-C	Alkaloid	HSV-2	NR	NR	NR	Larsen et al. (1994)
24.	*Bacillus licheniformis*	Exopolysaccharide	Polysaccharide	NR	NR	Marine	NR	Arena et al. (2006)
25.	*Geobacillus thermodenitrificans*	Exopolysaccharide	Polysaccharide	NR	NR	Marine	NR	Arena et al. (2009)
26.	*Nostoc flagelliforme*	Nostaflan	Polysaccharide	HSV, hCMV, H1N1	NR	Aquatic	NR	Kanekiyo et al. (2007)
27.	*Arthrospira platensis*	Anionic polysaccharides TK V3 and EPS	Polysaccharide	VACV	NR	Marine	^a^0.78 µg/ml	Radonić et al. (2011)
28.	*Pseudomonas* sp	Extracellular glycosaminoglycan and sulfated polysaccharide	Polysaccharide	HSV-1	NR	NR	1.4 µg/ml	Matsuda et al. (1999)
29.	*Pseudomonas* sp.	NR	Polysaccharide	HIV-1, HIV-2, HSV, H1N1, RSV, measles virus	NR	Marine	NR	Hashimoto et al. (1996)
30.	*Spirulina platensis*	Calcium spirulan	Polysaccharide	HSV-1, hCMV, H1N1, HIV-1, measles virus, mumps virus	Inhibition of replication	NR	0.86 μg/ml	Hayashi et al. (1996)
31.	*Streptomyces galilaeus*	Aclacinomycin	Oligosaccharide	Phage φX174 and λ	NR	NR	NR	Tanaka et al. (1983)
32.	*Arthrospira platensis*	Calcium spirulan	Polysaccharide	hCMV, HSV-1, HSV-2	Virus replication	Aquatic	0.142, 0.069, 0.333 mg/ml	Hernández-Corona et al. (2002)
33.	*Arthrospira platensis*	Spirulan-like substances	Phenolic acid	hCMV, HSV-1, HHV-6 and HIV-1	Inhibition of replication	Marine	NR	Hernández-Corona et al. (2002)
34.	*E. coli*	Resveratrol	Polyphenols	RSV	Immune response	GMM		Lim et al. (2011)
35.	*E. coli*	Apigenin	Lactone	HCV, PV	NR	GMM	NR	Lee et al. (2015)
36.	*E. coli*	Baicalein	Lactone	DENV-2, SARS-CoV2	Inhibition in attachment, genome replication and transcription step	GMM	NR	Li et al. (2019b)
37.	*E. coli*	Scutellarein	Lactone	SARS-CoV	Inhibition in genome replication and transcription step	GMM	NR	Li et al. (2019b)
38.	*E. coli*	Pinocembrin	Lactone	ZIKV	Inhibition in penetration	GMM	NR	Tao et al. (2018)
39.	*Streptomyces* sp.	Butenolides 1a, 1b, 2, 3, 4	Lactone	Adenovirus	NR	Marine	91 µM	Strand et al. (2014)
40.	*E. coli*	Rosmarinic acid	Lactone	HBV, HIV	Inhibition in genome replication and transcription	GMM	NR	Li et al. (2019d)
41.	NR	Caprolactins A and B	Lectin	HSV	NR	Marine	NR	Davidson and Schumacher, (1993)
42.	Microcystis *aeruginosa*	Microvirin	Lectin	HIV	NR	NR	NR	Huskens et al. (2010)
43.	*Streptomyces* sp.	Xiamycin	Terpenoid	HIV	HIV infection (blocks R5)	Endophyte	30 μM	Ding et al. (2010)
44.	NR	5α(H); 17α(H), (20R)-β-Acetoxyergost-8(14)-ene	Sterol	HSV1	NR	Marine	NR	Tong et al. (2012)
45.	*Pseudomonas* sp.	Extract	NR	Polio virus	NR	Marine	NR	Toranzo et al. (1982)
46.	*Vibrio marinus*	Extract	NR	hEV71	NR	Marine	NR	Magnusson et al. (1967)
47.	*Cyanobacter* sp.	Extract	NR	HSV1, VSV	NR	Marine	NR	Yasuhara-Bell et al. (2010a)
48.	*Pseudomonas* sp.	Extract	NR	HNV	NR	Aquatic	NR	Kamei et al. (1988)
49.	*Pseudomonas fluorescens*	Extract	NR	OMV, HNV	NR	Aquatic	NR	Kamei et al. (1992)
50.	*Polyangium* sp*. Myxococcus stipitatuss*	Thiangazole; phenalamide A1; Phenoxan	NR	HIV	HIV replication	NR	252, 386, 376 µM, respectively	Jurkiewicz et al. (1992)
51.	*Sorangium cellulosum*	Noricumazole A	NR	EVD	NR	NR	0.33 µM	Beck et al. (2016)
52.	*Labilithrix luteola*	Labindoles A; Labindoles B	NR	HCV	NR	NR	NR	Mulwa et al. (2018)
53.	*Actinobacteria* sp.	Formycin	NR	HNV, Polio virus VACV	NR	NR	NR	Hori et al. (1964); Takeuchi et al. (1966)
54.	*Actinobacteria* sp.	Coformycin	NR	HIV	NR	NR	NR	Sawa et al. (1967)
55.	*Actinobacteria* sp.	Oxanosin	NR	HIV	NR	NR	NR	Shimada et al. (1981); Nakamura et al. (1991)
56.	*Actinomycete* sp.	Benanomycins	NR	HIV	NR	NR	NR	Hoshino et al. (1989)
57.	*E. coli*	Violacein	NR	HSV	NR	GMM	NR	Jones et al. (2015)
58.	S. lavendulae	DNJ	NR	HBV	Using precursor, analog, metabolism inhibitors as regulators	GMM	NR	Jacob et al. (2007); Wu et al. (2019)
59.	*Actinomadura* sp.	Kijimycin	NR	HIV	NR	NR	5 µg/ml	Nakamura et al. (1981)
60.	*Streptomyces nashvillensis*	Bellenamin	NR	HIV	Reduce viral infectivity	NR	^a^0.2 µg/ml	Ikeda et al. (1996)
61.	*Streptomyces* sp.	Anthranoside C	NR	H1N1		Marine	171 µM	Che et al. (2018)
62.	*Streptomyces* sp.	Sarkomycin	NR	Phage f2	NR	NR	NR	Koenuma et al. (1974)
63.	*Streptomyces verticillus*	Siastatin B	NR	HNV	Sialidase activity of influenza virus	NR	NR	Umezawa et al. (1974)
64.	*Clostridium orbiscindens*	Desaminotyrosine	NR	H1N1	Through modulation of type I IFN	NR	NR	Steed et al. (2017)
65.	*Streptomyces* sp.	Antimycin A	NR	WEEV, FMV, LACV, EMCV	Inhibition of cellular mitochondrial electron transport chain	NR	4 nM	Raveh et al. (2013)
66.	*Trichodesmium erythraeum*	Debromoaplysiatoxin; Anhydrodebromoaplysiatoxin; 3-methoxydebromoaplysiatoxin	NR	CHIKV	NR	NR	^a^1.3 μM (Debromoaplysiatoxin) and 2.7 μM (3-methoxydebromoaplysiatoxin)	Gupta et al. (2014)
67.	Arthrospira fusiformis	Crude extracts	NR	HSV 1	Inhibits viral replication		NR	Sharaf et al. (2010)
68.	*Leptolyngbya* sp.	NR	NR	H1N1	Inhibition of replication	Aquatic	80–85 µg/ml	Silva et al. (2018)
69.	*M. aeruginosa, M. ichthyoblabe M. wesenbergii*	Extract	NR	H1N1	Protease inhibitor	NR	20.0–79.0 µg/ml	Zainuddin et al. (2002)
70.	*Nostoc sphaericum*	6-cyano-5-methoxy-12-methylindolo[2,3-α]carbazole and 6-cyano-5-methoxyindolo[3-α]carbazole	NR	HSV	NR	Marine	NR	Knübel et al. (1990)
71.	*Spirulina platensis*	Extract	NR	HIV-1	Inhibition of replication	NR	0.3 and 1.2 µg/ml	Ayehunie et al. (1998)
72.	*Spirulina maxima*	Extract	NR	HSV-2, PRV, HCMV, and HSV-1	NR	NR	^a^0.069, 0.103, 0.142, and 0.333 mg/ml	Hernández-Corona et al. (2002)

HIV, human immunodeficiency virus; HSV, herpes simplex virus; RSV, respiratory syncytial virus; hCMV, human cytomegalovirus; VACV, vaccinia virus; VSV, vesicular stomatitis virus; H1N1, Influenza A virus subtype H1N1; OMV, *Oncorhynchus masou* virus; HCV, Hepatitis C virus; WEEV, Western equine encephalitis virus; CHIKV, Chikungunya virus; LACV, La Crosse virus; EMCV, Encephalomyocarditis virus; EVD, Ebola virus disease; hEV71, Enteroviruses 71; HHV-6, Human Herpesvirus 6; NR, not reported;

a Indicates EC_50_ value of the compound.

**TABLE 3:** Antiviral bioactive compounds obtained from microalgae.

SL.	Microorganism name	Antiviral compound name	Group	Targeted virus	Mechanism of inhibition	Source of the microbe	EC_50_Value	References
1.	*Gyrodinium impudium*	Polysaccharide p-KG03	Polysaccharide	INF-A	Viral entry	Marine	0.19–0.48 μg/ml	Kim et al. (2012)
2.	*Porphyridium cruentum*	Highly sulfated polysaccharide	Polysaccharide	HSV-1, HSV-2, VACV	NR	Marine	NR	Huheihel et al. (2002)
3.	*Porphyridium purpureum*	Sulfated exopolysaccharide	Polysaccharide	VACV	Viral entry	Marine	0.65 µg/ml	Radonić et al. (2011)
4.	*Porphyridium* sp.	Sulfated polysaccharide	Polysaccharide	HSV-1, HSV-2, VZV	Viral infection	Marine	1 μg ml^−1^	Huleihel et al. (2001)
5.	*Rhodella reticulata*	Exopolysaccharide	Polysaccharide	Murine sarcoma and leukemia viruses	Inhibit early steps in the virus replication cycle	Aquatic	NR	Talyshinsky et al. (2002)
6.	*Gyrodinium impudicum*	Extracellular polysaccharide p-KG03	Polysaccharide	EMCV	Target receptors, intracellular machineries of replication	Marine	26.9 μg/ml	Yim et al. (2004)
7.	*Cochlodinium polykrikoides*	Extracellular sulfated polysaccharide A1 and A2	Polysaccharide	H1N1, RSV-A, RSV-B, Parainfluenza-2	NR	Marine	NR	Hasui et al. (1995)
8.	*Haematococcus pluvialis, Dunaliella salina*	Sulfated polysaccharide	Polysaccharide	HSV1	Viral attachment, intracellular replication	Marine	98.61 ± 3.78 μg ml^−1^; 85.34 ± 5.89 μg ml^−1^	Santoyo et al. (2010); Santoyo et al. (2012)
9.	*Chlorella autotrophica, Ellipsoidon* sp.	Sulfated polysaccharide	Polysaccharide	VHSV, ASFV	Inhibition of replication	Marine	NR	Fabregas et al. (1999)
10.	*Coccomyxa gloeobotrydiformis*	AEX	Polysaccharide	Infectious bursal disease virus (	Viral entry	NR	NR	Guo et al. (2017)
11.	*Coccomixa* sp.	A monogalactosyl diacylglyceride	Galactolipid	HSV 2	Inhibition of viral replication	NR	11 ± 0.42 μg ml^−1^ 11 ± 1.6 μg ml^−1^	Hayashi et al. (2019)
12.	Chlorella vulgaris	Pressurized liquid extracts (PLE)	Polysaccharide	HSV 1	Virucidal activity		61.05 μg/ml 80.23 μg/ml	Santoyo et al. (2010)
13.	*Staurastrum* sp*.*	Extract	NR	H1N1	Inhibition of replication	Aquatic	70–90 µg/ml	Silva et al. (2018)
14.	*Scenedesmus* sp.	Extract	NR	H1N1	Inhibition of replication	Aquatic	130 µg/ml	Silva et al. (2018)
15.	*Desmodesmus armatus*	Extract	NR	H1N1	Inhibition of replication	Aquatic	55–60 µg/ml	Silva et al. (2018)
16.	*Dunnliella primolecta*	Extract	NR	HSV	NR	Marine	NR	Ohta et al. (1998)

HIV, human immunodeficiency virus; HSV, herpes simplex virus; VZV, Varicella zoster virus; EMCV, Encephalomyocarditis virus; VHSV, viral haemorrhagic septicaemia virus; ASFV, Africa ASFV, Africann swine fever virus; H1N1, Influenza A virus subtype H1N1; VACV, Vaccinia; CVB3, Coxsackie B3 virus; PRV, pseudorabies virus; NR, not reported.

### Polysaccharides

Microbial polysaccharides (MPS), the biopolymers produced through microbial metabolic process, are widely found in bacteria, fungi and algae (Tables 1–3). The antiviral metabolites so far reported from algae are MPS (Table 3). However, bacteria and fungi produced a variety of MSM including MPS (Tables 1, 2). The advantages of MPS over plant polysaccharides include lack of seasonal, geographical, pest and diseases restriction; wide variety of sources as well as short production time (Chen and Huang, 2018). Some MPS are linear (cellulose, chitin, chitosan, pullulan, alginate, curdlan) and some are branched (dextran, levan, xanthan, scleroglucan, and in lesser degree gellan). Neutral (dextran, levan, pullulan, cellulose, scleroglucan and curdlan), anionic (alginate, xanthan, gellan), and cationic (chitin and chitosan) properties of these linear and branched MPS may make them suitable against a variety of viruses (Steed et al., 2017). Due to having diversified structural properties, the antiviral mechanisms of MPS are complex and diverse, and thus suitable for a variety of applications (Steed et al., 2017; Liu et al., 2020). The antiviral mechanisms of MPS include the inhibition of events involved in viral life cycle (attachment of virus to the host cell, penetration, genetic material and protein synthesis) and the improvement of the host immunity (Liu et al., 2020). However, the antiviral mechanism of many MPS is not yet known.

Recently, studies on derivatives of MPS are given priorities because chemical modification generates enhanced or new activities to MPS (Chen and Huang, 2018). The most common derivatives are sulfonated, phosphorylated and selenizated. The derivatives of MPS having lower or no toxicity even at higher concentrations offer broad prospects for treatment of viral diseases (Saha et al., 2012; Chen and Huang, 2018; Liu et al., 2020). The bioactive sulfated polysaccharide, p-KG03, obtained from *Gyrodinium impudicum* showed antiviral activity (EC_50_ = 26.9 µg/ml) against encephalomyocarditis virus (Yim et al., 2004) and inhibited H1N1 with an EC_50_ value of 0.19–0.48 μg/ml through interfering the viral entry into the host cell (Kim et al., 2012). Another sulfated polysaccharide isolated from red microalgae *Porphyridium* sp. showed impressive antiviral activity against Herpes simplex viruses types 1 and 2 (HSV 1, 2) and Varicella zoster virus (VZV) with IC_50_ 1 μg/ml (Huleihel et al., 2001). However, the same polysaccharide isolated from *Haematococcus pluvialis* showed similar inhibition rate against HSV-1 with IC_50_ 75 µg/ml concentration (Santoyo et al., 2012). Furthermore, a number of MPS obtained from various microalgae and bacteria showed promising antiviral activity with unknown mechanism of action against numerous viruses such as HIV1, HSV-1, HSV-2, Vaccina virus, Murine sarcoma and leukemia viruses, Influenza A and B viruses, RSV-A, RSV-B, parainfluenza-2, VHSV, ASFV, hCMV, VACV mentioned in Tables 2, 3.

### Peptides

Antiviral peptides (AVPs) obtained from natural sources are amphipathic and cationic nature. In addition, their hydrophobicity make them the promising drug candidate against enveloped viruses (Agarwal and Gabrani, 2020). The AVPs are reported from bacteria and fungi, however, not yet from algae (Tables 1, 2). Advantages of naturally produced microbial AVPs include high specificity and effectiveness, low toxicity and peptidase biodegradability, and low molecular weight (Boas et al., 2019). The AVPs can act at various stages of the viral life cycle through the suppression of viral gene expression. They can further prevent viral infection by many ways including inhibiting the viral particle or by competing for the receptor molecule in the host cell membrane and consequent adsorption, suppression of topoisomerase-mediated DNA-binding, DNA relaxation and formation of covalent complex (Galdiero et al., 2013; Heydari et al., 2021). Some of them can show activity by membrane destabilization of the virus (Rowley et al., 2004; Porotto et al., 2010). However, the mode of actions of most of the bacterial and fungal AVPs remains elusive (Tables 1, 2).

Sansalvamide A, a cyclic depsipeptide, isolated from marine *Fusarium* spp. showed antiviral activity against a poxvirus, molluscum contagiosum virus (MCV) by inhibiting the virus-encoded type-1 topoisomerase which is essential for MCV replication (Hwang et al., 1999). Simplicilliumtide J, a cyclic peptide, isolated from a deep sea derived fungal strain *Simplicillium obclavatum* EIODSF 020 and its analogues Verlamelin A and B showed very promising anti-HSV-1 activity with IC_50_ values of 15.6 μM (Liang et al., 2017). The cyclodipeptide diketopiperazines (DKPs) obtained from endophytic fungus *Aspergillus versicolor* exhibited anti-HSV activity through inhibition of NS3/4A protease with the IC_50_ value 8.2 μg/ml (Ahmed et al., 2017).

### Alkaloids

Alkaloids are structurally diverse secondary metabolites which have many therapeutic applications including antiviral activity (Cushnie et al., 2014). Most of the alkaloids used as therapeutics to treat human diseases are natural products of plants although plants are unreliable, low-yielding, expensive and unstable source (Bradley et al., 2020). However, several recent studies showed that a number of fungi produce alkaloids as an MSM acting against pathogenic microbes including viruses (Table 1) (Peng et al., 2019; Sadahiro et al., 2020; Raihan et al., 2021). Nevertheless, despite the potentiality, bacterial and algal sources for alkaloids are not yet reported. Although the mechanisms of all microbial alkaloids are not yet known (Table 1), a number of studies report that alkaloids inhibit DNA polymerase, Topoisomerase, reverse transcriptase and protein synthesis (Thawabteh et al., 2019; Bleasel and Peterson, 2020; Wink, 2020), and deactivate the viral infection by acting as DNA intercalator (Croaker et al., 2016). Six indole alkaloids isolated from mangrove derived fungus *Cladosporium* sp. PJX-41 showed antiviral activity against H1N1 with IC_50_ values 82–89 μM (Peng et al., 2013). Stachyflin, a sesquiterpenoidal alkaloid, obtained from *Stachybotrys* sp. RF-7260 by solid state fermentation showed a promising antiviral activity *in vitro* against H1N1 with IC_50_ value 0.003 μM (Minagawa et al., 2002). Three new isoindolinone-type alkaloids named chartarutines B, G, and H isolated from sponge derived fungus *Stachybotrys chartarum* has been shown as antiviral agents to inhibit replication of HIV-1 with the IC_50_ value 4.9–5.6 mM (Li et al., 2014). Recently, it has been shown that two aminosulfonyl group containing alkaloids named Scedapin C and scequinadoline A extracted from marine-derived fungus *Scedosporium apiospermum*, displayed significant anti-HCV activity by inhibiting HCV protease with the EC_50_ values 110.35 and 128.60 μM, respectively (Huang L.-H. et al., 2017). Huang Z. et al. (2017) further showed that a deep-sea-derived fungus *Aspergillus versicolor* SCSIO 41502 produced Aspergilols H and I which displayed anti-HCV activity with EC_50_ values 4.68 and 6.25 μM, respectively (Huang Z. et al., 2017).

### Polyketones

Many polyketides (derived from polyketones) isolated from microorganisms such as fungi and bacteria have been shown to inhibit the viral infection in a various way (Tables 1, 2). However, mechanisms of actions of most of the polyketones mentioned in this paper have to be elucidated. A group of polyketides are capable to inhibit viral replication. Two of such polyketides named as Alternariol and Balticolid isolated from *Pleospora tarda* and *Ascomycetous* strain exhibited potent antiviral activity with IC_50_ value 13.5 μM and 0.01 mg/ml, respectively (Shushni et al., 2011; Selim et al., 2018). While these polyketones inhibit viral replication, Sclerotiorin, another polyketone isolated from an endophyte *Penicillium sclerotiorum* essentially interferes with HIV-1 integrase and protease—two essential enzymes for maintaining the life cycle of the virus inside the host cell (Arunpanichlert et al., 2010). Furthermore, a group of polyketides namely sulfangolid C, soraphen F, spirangien B and epothilon D isolated from *Sorangium cellulosum* protects against HIV by interacting with the Acetyl-CoA carboxylate transferase enzyme (Martinez et al., 2013). Martinez et al. (2013) further found that Rhizopodin, derived from *M. stipitatus* is a potential antiviral agent although the mechanism of inhibition of the compound has not been elucidated. Another study found that marine microbe *Phoma* sp. produced Phomasetin which inhibited the HIV integrase, rendering it a potential drug compound against HIV (Singh et al., 1999). In fact, most of the microbial polyketides have been isolated till date is from the marine microorganisms. However, several fungi obtained from other sources are also reported to produce antiviral compounds having promising activity against DENV, ZIKV, Influenza virus, HCV and others (Table 1).

### Terpenoids

Terpenoids are one of the most abundant natural aromatic compounds mostly found in plants. However, some microorganisms can synthesize terpenoids (Yamada et al., 2015). Furthermore, microbial strains can be engineered to produce such terpenoids that have antiviral activities (Ma et al., 2020). The properties and medicinal uses of terpenoids are being continuously investigated by researchers for anticancer, antioxidant, antiviral, and anti-atherosclerotic activities (Nazaruk and Borzym-Kluczyk, 2015). Based on the number of carbon atoms, terpenoids are of different types (Wang et al., 2018). Different modes of actions of different terpinoids make them important against viral infection. For instance, ochraceopone A, isoasteltoxin, and asteltoxin obtained from antarctic fungus *Aspergillus ochraceopetaliformis* exhibited antiviral activities against the H1N1 and H3N2 influenza viruses by inhibiting viral growth through their protease suppression with IC_50_ values of >20.0/12.2 ± 4.10, 0.23 ± 0.05/0.66 ± 0.09, and 0.54 ± 0.06/0.84 ± 0.02 μM, respectively (Wang et al., 2016). Three sesquiterpenes named as (Z)-5-(Hydroxymethyl)-2-(6′)-methylhept-2′-en-2′-yl)-phenol, diorcinol, cordyol C were extracted from sponge-associated fungus *Aspergillus sydowii* which showed anti H3N2 activity with IC_50_ values of 57.4, 66.5 and 78.5 μM, respectively (Wang et al., 2014). In addition, a terpenoid compound called xiamycin derived from a bacterial endophyte (*Streptomyces* sp.) acts as anti-HIV agent through prohibition of beta-chemokine receptor CCR5 with IC_50_ value of > 30 μM (Ding et al., 2010). This class of metabolites can be produced in engineered fungi such *Saccharomyces cerevisiae* and *Yarrowia. Lipolytica* (Ma et al., 2020). Oleanolic acid is such a terpenoid produced from genetically modified *S. cerevisiae, which* inhibited genome replication and transcription of HCV (Zhao et al., 2018a). Another metabolite named betulinic acid produced from both *S. cerevisiae* and *Y. lipolytica* showed promising anti-HIV activity by inhibiting viral release from the host cell (Huang H. et al., 2019; Sun et al., 2019). Furthermore, a lot of terpinoids derived from fungi exhibited antiviral activity against numerous viruses such as H3N2, hEV71, H1N1, HBV, HIV, PRV, and DENV (Table 1).

### Quinone

Quinones are aromatic organic compounds and found ubiquitously in prokaryotes and eukaryotes. Quinones act through inhibition of electron transport as well as uncoupling of oxidative phosphorylation (Obach and Kalgutkar, 2018). Furthermore, they can act as inducers of reactive oxygen species and bioreductive alkylators of biomolecules, and suppress DNA function by interpolation into DNA (Roa-Linares et al., 2019). Quinones are used as antioxidant, antimicrobial, anticancer, anti-inflammatory, antitumor agents (El-Najjar et al., 2011; Teng et al., 2020). The coccoquinone A, an anthraquinone derivative, obtained from *Aspergillus versicolor* function as an anti-HSV agent with the EC_50_ value 6.25 µM (Huang Z. et al., 2017). Furthermore, 4-hydroxymethyl-quinoline isolated from myxobacteria *Labilithrix luteola* exhibited antiviral activity against HCV (Mulwa et al., 2018). Moreover, Alatinone, Emodin, and Hydroxyemodin, isolated from red alga *Liagora viscida* derived endophytic fungi *Penicillium chrysogenum* showed antiviral activity against HCV through inhibition HCV protease (Hawas et al., 2013). A citrinin dimer, seco-penicitrinol A obtained by coculturing of two marine algal-derived endophytic fungal strains *Aspergillus sydowii* and *Penicillium citrinum* showed inhibitory activity towards influenza neuraminidase *in vitro* with an IC_50_ value 24.7 µM (Yang et al., 2018). An anthraquinone derivatives called (‒)-2′R-1-hydroxyisorhodoptilometrin obtained from marine fungi *Penicillium* sp. OUCMDZ acted as an antiviral agent against HBV (Jin et al., 2018). Furthermore, some other promising antiviral quinone type compounds have been listed in Table 1.

### Sterols

Sterols, also known as steroid alcohols, found ubiquitously in numerous plant, animals as well as microorganisms are considered as common natural bioactive compounds (Hisham Shady et al., 2021). These natural compounds inhibit viral infection through suppression of lipid dependent viral attachment to the host (Hisham Shady et al., 2021). A highly oxygenated sterol compound called Cladosporisteroid B isolated from a sponge-derived fungus *Cladosporium* sp. acted as an antiviral agent against H3N2 with an IC_50_ value 16.2 µM (Pang et al., 2018). Another new compound named 3α-hydroxy-7-ene-6,20-dione containing a rare 3α-OH configuration and synthesized by the fungus *Cladosporium* sp. showed antiviral activity against the respiratory syncytial virus (RSV) with the IC_50_ value of 0.12 µM (Yu et al., 2018). Furthermore, an ergostane analogous metabolite named 3β-hydroxyergosta-8, 14, 24 (28)-trien-7-one isolated from the marine *Penicillium* sp. displayed broad-spectrum antiviral activities against HIV and H1N1 with the IC_50_ value of 3.5 and 0.5 µM, respectively (Li et al., 2019c).

### Pyrone

Pyrones, found as two isomers namely 2-pyrone and 4-pyrone, are comprised of an unsaturated six-membered ring with one oxygen atom and a ketone functional group (Teng et al., 2020). An endophytic *Fusarium equiseti* isolated from a marine brown alga *Padina pavonica*, secretes various extracellular metabolites in different media compositions (Hawas et al., 2016). When this endophytic fungus was cultivated in biomalt-peptone medium, it produced 12 known metabolites of diketopeprazines and anthraquinones which were very potent anti-HCV (HCV protease inhibitor) agent with an IC_50_ from 19 to 77 μM, and the most potent anti-HCV compound in this condition was Griseoxanthone C with IC_50_ value of 19.8 μM (Hawas et al., 2016). However, the same fungus released nine different types of anti-HCV agents with IC_50_ value of 10–37 μM in the presence of Czapek’smedia, and the most potent anti-HCV compound was ω-hydroxyemodin with IC_50_ value of 10.7 μM (Hawas et al., 2016). “One strain many compounds” (OSMAC) has been proposed as a very effective approach to discover novel bioactive compounds (Pan et al., 2019). With the OSMAC approach, a coastal saline soil-derived fungus *Aspergillus iizukae* produces different antiviral compounds namely Methyl-(2-chloro-l,6-dihydroxy-3-methylxanthone)-8-carboxylate; methyl-(4-chloro-l,6-dihydroxy-3-methylxanthone)-8-carboxylate; methyl-(4-chloro-6-hydroxy-1-methoxy-3-methylxanthone)-8-carboxylate; methyl-(6-hydroxy-1-methoxy-3-methylxanthone)-8-carboxylate; 4-chloro-1,6-dihydroxy-3-methylxanthone-8-carboxylic acid; and 2,4-dichloro-1,6-dihydroxy-3-methylxanthone-8-carboxylic acid (Kang et al., 2018). Among these compounds, methyl-(4-chloro-l,6-dihydroxy-3-methylxanthone)-8-carboxylate exhibits strong antiviral activities against H1N1, HSV-1, and HSV-2 with IC_50_ values 44.6, 21.4, and 76.7 µM, respectively. However, the other compounds show week antiviral activity (Kang et al., 2018). A marine bacteria *Streptomyces youssoufiensis* can produce antiviral violapyrones (VLPs) Q–T through heterologous expression of the type III polyketide synthase (PKS) gene *VioA* (Hou et al., 2018). The antimicrobial activity of violapyrones mainly depends on the modification of 4-OH (methylation/non-methylation) (Teng et al., 2020). The compound showed antiviral activity in methylated condition but it showed anti-MRSA (Methicillin-resistant *Staphylococcus aureus*) activity in non-methylated condition with losing antiviral activity. The results support the notion that methylation at 4-OH of these compounds enhanced anti-virus activity but reduced anti-MRSA activity (Hou et al., 2018).

### Polyphenol

Polyphenols or phenolic compounds are one of the prominent bioactive compounds found as secondary metabolites in plants and microorganisms (Othman et al., 2019; Carpine and Sieber, 2021). For instance, a soil fungus *Exophiala pisciphila* produces a novel dimeric 2,4-dihydroxy alkyl benzoic acid which exhibits anti-HIV activity by inhibiting integrase, a most crucial enzyme for HIV pathogenesis and is one of the most promising drug targets for anti-retroviral therapy (Ondeyka et al., 2003). Some antiviral polyphenol compounds have been produced through genetically engineered *Saccharomyces cerevisiae, E. coli*, *Penicillium brevicompactum*, *Streptomyces avermitilis*, *Streptomyces lavendulae*, and *Yarrowia lipolytica* (Ma et al., 2020). These prominent bioactive compounds exhibit antiviral activities through numerous mechanisms such as inhibition of viral attachment, penetration, genome replication and transcription as well as translation and viral assembly (Tables 1, 2) (Ma et al., 2020).

### Lectin, Lipid, Lignan

A unique 95 amino acid long antiviral lectin obtained from a cyanobacterium *Scytonema varium* inhibits HIV attachment to the host cell through binding with the viral coat proteins gp120, gp160, and gp41 with EC_50_ values ranging from 0.3 to 22 nM (Bokesch et al., 2003). In addition, two prominent antiviral compounds namely cyanovirin-N and agglutinin obtained from cyanobacterium *Nostoc ellipsosporum* and *Oscillatoria agardhii*, respectively act as anti-HIV agents. The former compound inhibits viral attachment by binding with gp120 and the later one inhibits viral replication (Boyd et al., 1997; Sato et al., 2007). Furthermore, a glycolipid derived from cyanobacterium showed remarkable antiviral activity against HIV-1 (Gustafson et al., 1989). Phenylpropanoid units containing compound such as podophyllotoxin of endophytic *Fusarium oxysporum* isolated from *Juniperus recurva* showed anti-HIV activity (Kour et al., 2008). Furthermore, lots of bioactive compounds show antiviral activity against various viruses such as Human cytomegalovirus; HIV, H1N1, HSV, DENV, Enterovirus 71, ZIKV, RSV, HBV, HCV, Western equine encephalitis virus, and Pseudorabies virus (Tables 1, 2).

## Potential Microbial Metabolites Against SARS-CoV-2

No newly developed specific drug has been approved by the WHO, FDA or any other global regulatory body to treat SARS-CoV-2. However, some drugs for other diseases have been approved for emergency usage during the pandemic situation (Hakim et al., 2021). For instance, the microbial-derived anti-parasitic drug ivermectin (Patridge et al., 2016) has been approved by the FDA to treat COVID-19 patients. Nevertheless, the time was not also enough to discover specific drug against SARS-CoV-2. However, research is going on globally to find drug against SARS-CoV-2 either from microbial or plant sources. A semisynthetic pentacyclic sixteen-membered lactone obtained from the soil bacterium *Streptomyces avermitilis*, has been found *in vitro* as inhibitor of SARS-CoV-2 replication (Caly et al., 2020). To find anti-SARS-CoV-2 drug from either microbial or plant sources, mostly *in silico* studies have been done. *In silico* screening, molecular docking, ADMET (Absorption, Distribution, Metabolism, Elimination, and Toxicity) prediction and molecular dynamic simulation (MDS) carried out by a number of studies predicted several phytocompounds as the potential inhibitors of SARS-CoV-2 and could be candidates to the discovery of novel drugs for the treatment of COVID-19 (Basu et al., 2020; Bhuiyan et al., 2020; Choudhary et al., 2020; Prasanth et al., 2020; Puttaswamy et al., 2020; Zhang et al., 2020; Chandra et al., 2021; Prasanth et al., 2021; Sankar et al., 2021). A study screened six potential candidates (Citriquinochroman, Holyrine B, Proximicin C, Pityriacitrin B, (+)-Anthrobenzoxoconone, and Penimethavone A) as anti-SARS-CoV-2 from >24,000 natural microbial compounds (Sayed et al., 2020). Docking andMDS analysis suggests that these microbial metabolites are potential inhibitor of protease involved in the host-SARS-CoV-2 interaction. However, experimental validation is required for the hypothesis derived from the *in silico* studies of plant and microbial metabolites.

Since the outbreaks of SARS in 2002/2003, MERS in 2012 and the COVID-19 pandemic in 2019/2020 (all caused by β-coronaviruses), different antiviral natural compounds have been tested against coronaviruses, such as remdesivir, ribavirin or herbacetin (Cherian et al., 2020). A numbers of microbial metabolites have been discussed in the aforementioned section to show antiviral activity including viral respiratory infections. Most of the microbial metabolites listed in Tables 1–3 are experimentally reported. Some of these metabolites especially those that show activity against viral respiratory infection can be potential for repurposing drugs against SARS-CoV-2. However, it would be worth for the researchers to elucidate the mechanism of actions of all antiviral microbial metabolites. Therefore, it will be interesting to perform docking and MDS of these microbial metabolites against proteins of SARS-CoV-2 and/or humans to predict their mechanism of actions, and finally experimentally validate the prediction of the *in silico* study. Metabolites from probiotic bacteria and/or gut microflora have been suggested to prevent viral respiratory infections including COVID-19 (Chen J. et al., 2021; Gautier et al., 2021). Probiotic bacterial metabolites such as butyrate, desaminotyrosine, and secondary bile acid may be transported to the lung via the circulation and could prevent viral respiratory infections by inhibiting viral replication or improving the immune response against viruses (Tiwari et al., 2020; Gautier et al., 2021). However, extensive studies are required to conclude the benefits of metabolites from probiotic bacteria and/or gut micro flora in COVID-19.

## Advantages of the Microbial Source for Antiviral Metabolites

Currently, researchers are focusing on natural bioactive compounds to control viral infections that are considered as the main cause for human death worldwide (Akram et al., 2018). They are designing natural broad-spectrum antiviral agents by targeting a common pathway but essential for functions in many viruses (Vigant et al., 2015). The sources of natural bioactive compounds are plants, animals and microorganisms. However, as the leading producers of essential natural bioactive compounds, microorganisms are preferred more. Microorganisms are advantageous over other natural sources such as plants and animals due to their certain unique characteristics. Most of microorganisms are available as a wide range of genetically specified strains, fast growth, high density, high production rate, efficient secretion, easy handling and propagate, and can be easily manipulated (Singh et al., 2017). Microorganisms in general act as the source of essential natural product having the advantage of viable and sustainable production of secondary metabolites by large scale fermentation with reasonable cost (Waites et al., 2009; Sun X. et al., 2015). Furthermore, microorganisms can be grown at large amount in a small space such as in a fermenter under a wide range of environmental conditions for production of MSM of versatile groups. However, plants and animals need large space and longer period for cultivation, and are not amicable to versatile environmental conditions and/or metabolic engineering is technically challenging to plants and animals (Tatsis and O’Connor, 2016).

Metabolic and genetic engineering can easily be applied to microorganisms. Genomic information of a microbe makes it easy to apply metabolic engineering to scale up the production and/or modify the natural bioactive compound (Ma et al., 2020). Modified natural bioactive compounds may be suitable to get rid of drug resistance of viruses with their high genetic variability, and microbes are the most preferable candidates in this case (Lin et al., 2014). Furthermore, metabolic engineering to contrive the microbial cellular metabolic machinery and the fermentation technology to scale up the production has introduced a low-cost microbial system for large scale production of many natural bioactive compounds including antiviral agents (Liu and Nielsen, 2019; Pham et al., 2019; Ma et al., 2020). For instance, Violacein is a bis-indole pigment produced by several Gram-negative bacterial species by the vioABCDE operon (Choi et al., 2015). Due to antimicrobial (antibacterial, antiviral and antifungal) properties, this compound has become an interesting target for metabolic engineering strategy. Recently, the *Y. lipolytica* chassis strain was engineered for increased production of this compound. Introduction of five genes of bacterial vioABCDE operon and overexpression of endogenous anthranilate synthase 2 and 3 of *Y. lipolytica* increased violacein production 2.9 fold in comparison with the control (Zheng et al., 2020). Thus, heterologous synthesis of many antiviral compounds in genetically engineered microbes which are safer and economically beneficial offers some significant advantages over plant extraction and chemical synthesis (Ma et al., 2020). However, expression of the biosynthetic pathways for production of particular compounds in microbial factories may not be cost-effective sometimes due to mainly complexity of the pathways involving a number of enzymatic steps (Pandey et al., 2016; Yang D. et al., 2020). Introduction of a number of foreign proteins in a single microbial cell may lead to unwanted interaction between genetic factors and overload of the cell capacity, resulting in decreased microbial growth and low yields of the metabolite (Johnston et al., 2020). In this case, coculturing might be a highly promising approach to overcome these complexities with high yield. Furthermore, recombinant DNA technology used for large scale industrial production of bioactive compounds is feasible in microbial systems. The advancement of recombinant DNA technology has opened new windows for development of bioactive natural products and biologics (Pham et al., 2019; Ma et al., 2020). However, the choice of microbial host cells is very crucial for production of natural and recombinant products. Different tools and strategies for engineering host cells as microbial cell factories for production of natural bioactive compounds and recombinant products have been discussed elsewhere (Pham et al., 2019; Ma et al., 2020).

## Future Prospects and Conclusion

The microbial source and system for antiviral natural bioactive compounds is attracting the researchers due to its advantages over plant and animal sources. Consequently, the demand of antiviral microbial metabolites is gradually increasing because the plant extraction and chemical synthesis cannot meet the global demand due to environmental, longer time and economic concerns. Microbial fermentation technology and metabolic and genetic engineering in microbial cells provide an alternate for scalable synthesis of these compounds. The global market value for MSM including antiviral agents was 277 billion USD in 2015, which is predicted to be 400 USD by 2025 (Park et al., 2019). Again, about 77% of FDA approved antimicrobial agents are produced from microbial sources, indicating microbial bioactive compounds as the pivotal source of antimicrobial drugs (Patridge et al., 2016). Therefore, antiviral microbial metabolites may pose great possibility in the field of pharmaceutical research and commercialization in near future. However, the vast diversity of antiviral microbial natural products yet requires extensive research and evaluation to find out the specific bioactive compounds with desired medicinal properties. Hence, from selection of appropriate microorganisms to formulation of drugs from their metabolites is a long term, expensive process that deserves relentless efforts and continuous exploration (Park et al., 2019).

Despite of some drawbacks such as final product purification and structural identification, microbial metabolite is still the unparalleled source of plenty of novel antiviral drug compounds (Park et al., 2019; Ma et al., 2020; Yi et al., 2020). Advancement of OMIC sciences (genomics, proteomics, metabolomics and so on) and gene based molecular approaches such genome editing, protein engineering and mutagenesis may offer more convenient drug design. Metabolomics being an emerging area in OMICs play pivotal roles in screening of lead compound, identifying drug target and assess bioactivity, potentiality and toxicity of the metabolites. Therefore, metabolomics in addition to proteomics that allows the structural and functional evaluation of the protein or antigenic compound targeted for the drug might be a great demand now-a-days in the term of drug designing and pharmacological research (Jain, 2004; Wishart, 2016). Furthermore, the most recent genome editing tool known as CRISPR (Clustered Regularly Interspaced Short Palindromic Repeats) can also be implemented in order to make desired change in the genome, especially while designing recombinant proteins in microbial cells to explore novel antiviral drugs (Liu et al., 2016). Similar site-specific gene editing by Zinc-finger nucleases (ZFNs) and transcription activator like effector nucleases (TALENs) possess great potentiality to be used in therapeutic purpose (Gaj et al., 2013). Therefore, OMICs and gene editing approaches collectively can be feasible in achieving the desired goal in screening and modifying microbial metabolites for antiviral drugs. Another efficient approach is microbial genome mining which comes with an outstanding opportunity of evaluating activity of the silent gene and discovering novel metabolites with the assistance of the information from genome sequencing (Bachmann et al., 2014). It also enables the understanding of biochemical pathways taking place inside the microbial cell, thus allowing the potential antiviral drug compounds to be discovered and analyzed (Fields et al., 2017; Xia, 2017). Furthermore, in the near future, metabolic engineering will contribute a lot to the discovery and development of antiviral drugs from microbial metabolites. Microbial system is becoming popular for expressing heterologous antiviral bioactive compounds. However, it paves challenges to the researchers to design and express the multiple enzymatic pathways involved in biosynthesis of antiviral bioactive compounds.

A wide array of plant-based secondary metabolites show promising antiviral activity against coronaviruses (Bhuiyan et al., 2020). Microbial biotechnology may contribute to large scale production of antiviral plant secondary metabolites or to get novel pharmaceutically active metabolites. However, many of the antiviral microbial metabolites included in this study are synthesized by endophytes. Therefore, the promising plant-based metabolites can be achieved through the screening of endophytic organisms of the targeted plant because various endophytic bacteria and fungi have the ability to produce the same or similar compounds as their host plants (Xu et al., 2009; Gouda et al., 2016; Raihan et al., 2021). For example, taxol, a billion dollar anticancer drug, initially produced by *Taxus brevifolia* and now it is produced from its endophyte *Taxomyces andreanae* (Stierle et al., 1993). Similarly, camptothecin, podophyllotoxin, hypericin and azadirachtin, are produced both by the endophyte and its host plant (Kusari and Spiteller, 2011; Bhalkar et al., 2016). Therefore, metabolites of endophytic microorganisms could be an emerging source of antiviral bioactive compounds (Schulz et al., 2002; Xu et al., 2009; Gouda et al., 2016; Raihan et al., 2021). Finally, researchers should pay attention to research with microbial metabolites using the approaches aforementioned to combat against catastrophic viral infections including COVID-19 and potential outbreaks of future viral pandemic and/or epidemics. For this, it is necessary to adopt initiatives to conduct systematic longitudinal studies by applying available and newly discovered microbial metabolites against catastrophic viruses including SARS-CoV-2.
